# Measure method of effective diffusion in gas oscillating in channels of variable radius or porous medium

**DOI:** 10.1016/j.mex.2021.101552

**Published:** 2021-10-14

**Authors:** Denis Polezhaev, Victor Kozlov, Antonio Viviani

**Affiliations:** a Perm State Humanitarian Pedagogical University, Laboratory of Vibrational Hydromechanics, Perm, Russia; b Università della Campania “Luigi Vanvitelli”, Dipartimento d'Ingegneria, Aversa (CE), Italy

**Keywords:** Molecular diffusion, Effective diffusion, Oscillations, Porous medium, Tortuosity

## Abstract

The paper discusses a new technique for measuring the diffusion coefficient of vapor of a volatile fluid in air in long straight channels or porous media. The proposed experimental technique is universal and allows a) determining the coefficient of molecular diffusion of vapor of a volatile liquid in air; b) calculating the coefficient of effective diffusion of vapor in oscillating air. The proposed technique was tested in the study of the diffusion of 2-propanol vapor in air at rest and oscillating air in a channel of variable radius and a porous medium consisting of randomly packed hard spheres of equal diameter and was found to be relevant. Initial experiments with a porous medium show that the proposed experimental technique can also be used to estimate the diffusive tortuosity of porous media. The results of studies, finished and planned, are useful for understanding the physical processes taking place in various technical operations including wood and food drying, drug delivery, etc.

• The new experimental technique provides accurate measurement of the molecular diffusion coefficient of vapor of a volatile fluid in air.
• The experimental setup allows measuring the enhanced mass transfer of vapor of a volatile fluid in oscillating air in a straight channel of constant/variable radius and a porous medium.
• The additional advantage of the present technique is that it enables the estimation of the diffusive tortuosity of a porous medium.

## Graphical abstract

**Figure:**
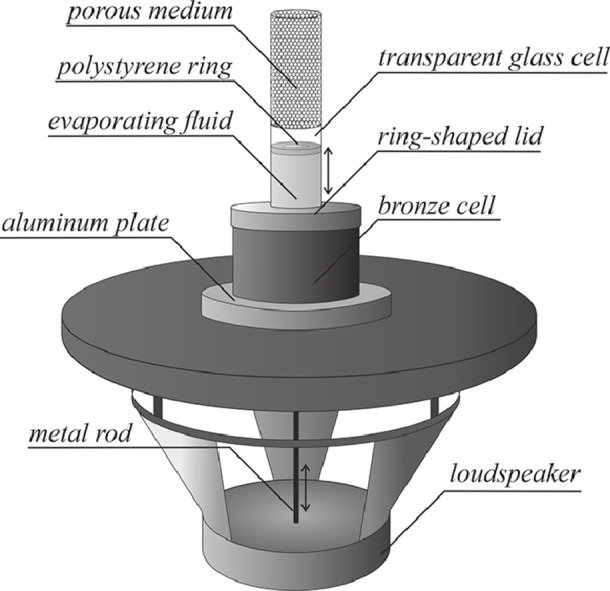

## Nomenclature

*b_air_p_*: amplitude of air oscillations in a porous medium, [m]
*b_air_r_*: amplitude of air oscillations in a channel of radius *r*, [m]
*b_f_*: amplitude of fluid oscillations, [m]
*D*: coefficient of molecular diffusion, [m^2^·s^−1^]
*D_eff_*: coefficient of effective diffusion, [m^2^·s^−1^]
*D_ch_*: experimental value of molecular diffusion coefficient of 2-propanol vapor in air in a straight channel, [m^2^·s^−1^]
*D_p_*: diffusion coefficient of vapor in air in a porous medium, [m^2^·s^−1^]
*d*: diameter of spherical particles composing the porous medium, [m]
*d*_1_: inner diameter of the bronze cell, [m]
*d*_2_: external diameter of the bronze cell, [m]
*d*_3_: inner diameter of the metal ring-shaped lid, [m]
*dh_f_*/*dt*: velocity of free surface of evaporating fluid, [m·s^−1^]
*dm*/*dt*: rate of mass change of evaporating fluid, [kg·s^−1^]
*f*: frequency of liquid and air oscillations, [Hz]
*h*_1_: height of the bronze cell, [m]
*h*_2_: thickness of the metal ring-shaped lid, [m]
*h*_3_: height of the transparent glass cell, [m]
*L*: height of a channel or thickness of the layer of porous medium, [m]
λ: dimensionless parameter affecting the diffusive tortuosity
μ_0_: molar mass of 2-propanol, [kg·mol^−1^]
μ*_air_*: molar mass of air, [kg·mol^−1^]
ν: kinematic viscosity of air, [m^2^·s^−1^]
*P*: porosity
*Pe* = 2π*fb_air_d*/*D*: Peclet number
*p*_0_: vapor pressure of 2-propanol, [Pa]
*R*: radius of aluminum plate, [m]
*R_g_*: ideal gas constant, [J·K^−1^·mol^−1^]
*r*_1_: inner radius of the transparent glass cell, [m]
*r*_2_: external radius of the transparent glass cell, [m]
ρ: density of air saturated with 2-propanol vapor, [kg·m^−3^]
ρ_0_: density of vapor 2-propanol, [kg·m^−3^]
ρ*_air_*: density of dry air, [kg·m^−3^]
ρ*_f_*: density of liquid 2-propanol, [kg·m^−3^]
*T*: temperature of air and 2-propanol, [K]
τ: coefficient of diffusive tortuosity
τ*_exp_*: experimental value of diffusive tortuosity of randomly packed monosized hard spheres

Specification table

**Table:** 

Subject Area	Physics and Astronomy
More specific subject area	Heat and Mass Transfer
Method name	Effective diffusion coefficient measurement technique
Name and reference of original method	N/A
Resource availability	N/A

### Method detailed

#### Introduction

The study of enhanced heat and mass transfer is of great interest because of the large number of relevant engineering applications. For instance, many scholars have conducted lots of researches on the design of high-efficiency heat exchangers [1]. The original method to enhance heat transfer is demonstrated in the numerical study of steady air flow in a sinusoidal channel filled with porous foam containing a phase change material (PCM) [2]. In a sinusoidal channel, the air flow regime is efficient to provide rapid cooling and further solidification of a liquid PCM in a porous foam. Due to the solidification of PCM, the heat is released and the air becomes warmer.

For decades, mechanical oscillations at a frequency above the threshold of human hearing (ultrasound) have been implemented as an effective tool to enhance mass transport of moister for wood [3] and food [4] drying. According to numerous studies of drying, the enhanced mass transport is associated with cavitation, “sponge effect”, deformation of the porous solid material, etc. Also, the therapeutic application of ultrasound enables the efficient delivery of drugs into and through the skin [5]. It has been commonly believed that ultrasound effects on tissues are mainly through cavitation, thermal effect, and steady streaming. Despite a large number of studies, it is still unknown which of the mechanisms involved in the ultrasonic attenuation in porous media are dominant.

It is worth mentioning that steady streaming is also an inherent part of thermal vibrational convection in rotating and oscillating cavities: Under high-frequency oscillations, the non-zero steady flow originates and impacts the mixing of non-isothermal liquid [6].

An alternative way to increase heat or mass transfer is to excite oscillations of a fluid (or gas) in a stationary container. For example, longitudinal air oscillations increase the mass transfer of a contaminant by several times due to Taylor dispersion in a long straight tube [7, 8]. The other direction of research relies on oscillatory fluid (or gas) flow inside a channel of alternating radius. Nishimura et al. [9] studied oscillatory fluid flow in a symmetric sinusoidal wavy walled channel by using an electrochemical technique and observed unsteady vortices in each section of the channel (the flow patterns were visualized by the aluminum dust method). The vortical flow is the result of nonlinear interaction between curved walls and oscillatory flow and appears and disappears within the oscillation cycle. The effect of unsteady vortices tends to diminish when the ratio of a fluid amplitude to the length of a channel section decays. In the limit of small amplitudes, unsteady vortices vanish but instead of them steady vortices are excited.

Recently, Kozlov and Polezhaev experimentally studied the effect of air oscillations on the diffusion rate of vapor in air in a channel of variable radius [10]. They discovered a novel phenomenon associated with the significant enhancement of longitudinal mass transfer due to steady vortical flows. To do that, the authors designed and assembled an experimental setup and developed a new experimental technique. In the experiments, the liquid evaporated near the bottom end of the channel of variable (or constant) radius. The emerging vapor diffused through the column of air at rest or oscillating air. In another recent work, Kozlov and Polezhaev used the developed experimental technique to study the effect of oscillations on the diffusion of vapor in air in porous media [11]. The crucial idea of the two mentioned papers is to study the enhanced mass transfer of species in the presence of steady streaming in order to get a better understanding of phenomena related to drug delivery, wood and food drying, etc. The present paper focuses on methods that were used in those studies to measure a) coefficient of molecular diffusion of vapor in air, b) coefficient of effective diffusion of vapor in oscillating air, and c) diffusive tortuosity of porous media.

#### Experimental setup

**Fig. 1:**
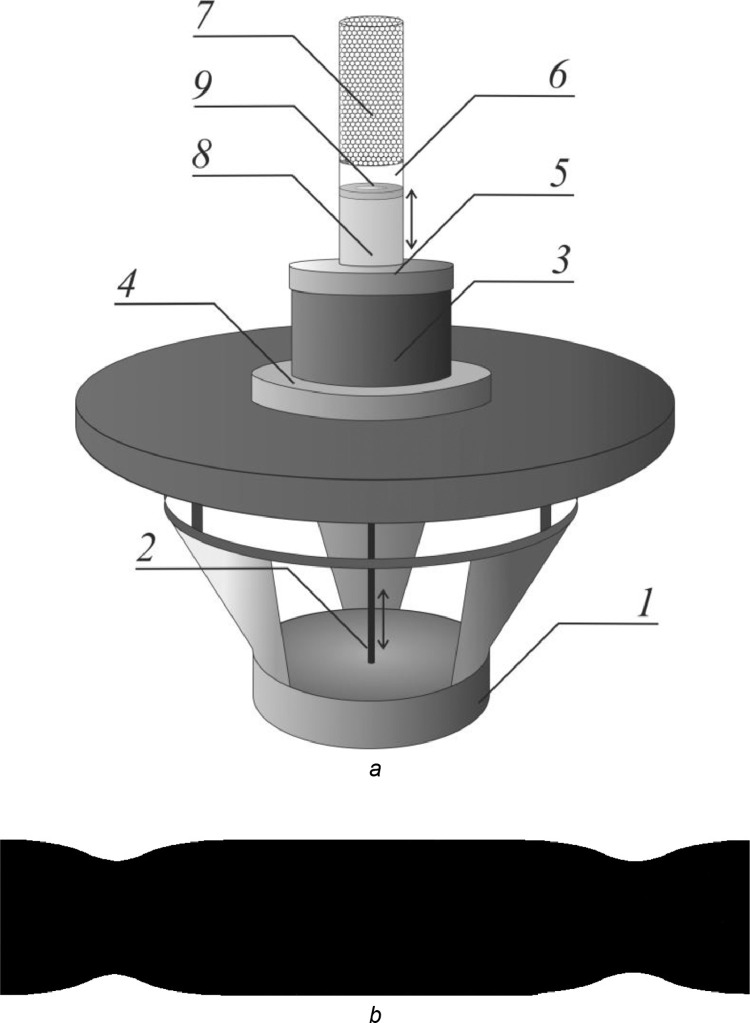
Schematic view of the experimental setup to measure the coefficient of effective diffusion of vapor of fluid in oscillating air in a porous medium (*a*) and the channel of variable radius (*b*).

The experimental setup is designed to measure the coefficient of effective diffusion of vapor of a volatile fluid in oscillating air in a straight channel of the constant or variable radius or a porous medium. The longitudinal oscillations of the air column in a channel (or in a porous medium) are generated by a loudspeaker *1* (Fig. 1*a*). In the experiments, we use a loudspeaker, which is controlled by a digital generator ZETLab [12]. The output voltage of the digital generator is too weak to provide mechanical oscillations of the loudspeaker of sufficient amplitude so that the electrical signal of the generator is transmitted to the amplifier Digisynthetic DP3200 and then to the loudspeaker.

The metal rod *2* of diameter 3 mm is glued to the center of the loudspeaker dust cap. The upper end of the rod goes through a hole in the center of the rubber membrane (not shown in Fig. 1). The membrane is attached to the rod using two nuts and two collars that are screwed on both sides of the membrane. To prevent unwanted evaporation of volatile fluid through the hole in the membrane, the nut and the collar on the loudspeaker side are glued to the rod.

The membrane transmits oscillations from the loudspeaker to the fluid inside the bronze cell *3* with the following dimensions: inner diameter *d*_1_ = 45 mm, external diameter *d*_2_ = 65 mm, and the height *h*_1_ = 30 mm. The cell is surrounded by a helically coiled heat exchanger (not shown in Fig. 1). To ensure better contact, the bronze cell and the heat exchanger are soldered to each other. The water in the heat exchanger is heated by the circulation thermostat LOIP LT-316. The thermostat allows maintaining the temperature of a volatile liquid inside the bronze cell with an accuracy of 0.1°C.

The rubber membrane is a circle with a diameter equal to the external diameter of the bronze cell. The membrane is clamped between the bronze cell and the aluminum plate *4* of radius *R* = 100 mm. The plate is fixed to the bronze cell with 6 bolts located around the circumference. For this purpose, six threaded holes are made inside the bronze cell. The bronze cell is filled with a volatile fluid and covered with a metal ring-shaped lid *5* of external diameter approximately equal to *d*_2_, inner diameter *d*_3_ = 25 mm, and thickness *h*_2_ = 10 mm. The lid is fixed to the bronze cell with six bolts located around the circumference. A rubber ring-shaped gasket is inserted between metal surfaces to prevent unwanted evaporation of volatile fluid. For this purpose, a groove for the rubber gasket is made in the upper end of the bronze cell.

There is a hole in the center of the lid for the transparent glass cell *6*. The transparent cell is used to measure the evaporation rate of the fluid: the upper end of the cell is open, as the fluid evaporates, the height of the fluid column decreases. To prevent unwanted evaporation of a fluid, the gap between the aluminum lid and the glass cell is filled with glue. The transparent glass cell is of inner radius *r*_1_ = 11.5 mm, external radius *r*_2_ = 12.5 mm, and height *h*_3_ = 100 mm.

When the experimental setup is used to study the diffusion of vapor of a volatile fluid in a channel of variable radius, the upper end of the glass cell is covered with a rubber stopper, in the center of which there is a hole with a diameter equal to the diameter of the channel flange. When the diffusion through a porous medium is studied, a grid is installed inside a glass cell, onto which glass beads are dropped. The thickness of porous medium *7* is chosen so that the randomly packed beads reach the edge of the glass cell.

### Experimental technique

The experiments are conducted using 2-propanol: density of liquid 2-propanol ρ*_f_* = 783 kg/m^3^, density of vapor 2-propanol ρ_0_ = 0.124 kg/m^3^, air kinematic viscosity ν = 15.4 mm^2^/s, and the diffusivity of vapor in air *D* = 9.8 mm^2^/s at temperature 23°C.

Above the free surface of a volatile fluid, the air is saturated with its vapor; its density can be calculated by the following equation

(1) $$\rho =\rho_{\text{air}}+\left(\mu_{0}-\mu_{\text{air}}\right)\left(p_{0}/R_{g}T\right),$$

here ρ_air_ is the density of dry (ambient) air, μ_air_ = 29 g/mol is the molar mass of air, μ_0_ = 60.1 g/mol is the molar mass of 2-propanol, *p*_0_ is the vapor pressure of 2-propanol at ambient temperature *T*. Note that, in the process of deriving Eq. (1) we neglected the small error due to non-ideal air compressibility. Since the molar mass of 2-propanol is larger than the molar mass of air, the second term in Eq. (1) is positive, and ρ > ρ_air_. Near the top edge of the channel of variable radius or porous medium, the vapor concentration equals 0, so that air density is equal to the density of ambient air. The density of vapor saturated air near the lower boundary of the channel (or porous medium) is larger than the density of atmospheric air, thus gas in the vertical channel is stably stratified and convective mass transfer is absent. In the absence of other mechanisms, the vapor is transported by molecular diffusion only.

The frequency of the imposed fluid oscillations varies in the range *f* = 0 – 150 Hz. The amplitude of the fluid oscillations is measured with the use of DSLR Canon EOS 60D with Canon Lens EF-S 35 mm f/2.8 IS STM Macro Led, placed in front of the transparent glass cell and focused on the interface between the fluid and air. The interface is illuminated by white LEDs. The resolution of the camera sensor is 18 MP (5184 × 3456 pixels). Taking into account that the size of the imaging area is around 45 mm × 30 mm, one can estimate that resolution of the digital image is approximately 115 pixels per mm.

The surface of oscillating fluid *8* in transparent glass cell is unstable to the onset of unwanted oscillations, for instance, Faraday ripples. These ripples make it impossible to measure accurately the amplitude of fluid oscillations. To prevent the rise of unwanted oscillations, the free fluid surface is covered by polystyrene ring *9* with an inner diameter of 16 mm, an external diameter of 22 mm, and a height of 4 mm. The upper surface of the ring is coated with the fluid so that it does not block the evaporation. The amplitude of fluid oscillations *b_f_* varies from 0 to 1 mm in the studied range of frequencies.

The vapor diffusion rate depends not so much on the amplitude of fluid oscillations as on the amplitude of air oscillations in a channel or pores. The amplitude of air oscillations can be calculated using the condition of equality of the air flow rate in the transparent glass cell and the channel or porous medium.

Consider the method to calculate the amplitude of air oscillations in narrow and wide sections of the channel of various radius (Fig. 1*b*). In the transparent cell, the air volume displaced by the oscillating fluid is equal to 4π*r*_1_^2^*b_f_*. In the channel of various radius, the volume of air passing through the cross-section of radius *r* is equal to 4π*r*^2^*b_air_r_*. Due to the incompressibility of air, the condition 4π*r*_1_^2^*b_f_* = 4π*r*^2^*b_air_r_* is satisfied, i.e.

(2) $$b_{air\_r}=b_{f}{\left(r_{1}/r\right)}^{2}.$$

Now consider the method to estimate the amplitude of air oscillations in pores. Preliminarily, we need to calculate the porosity *P* of randomly packed hard spheres, which is the ratio of the total volume of air pores to the total volume of the porous medium. The porosity of the randomly packed spheres depends, among others, on the ratio of spheres to cell diameter (for example, [7]). In this regard, the measurement of porosity should be carried out in a pipe of the same diameter as the experiments on the study of vapor diffusion. Although the size and shape of voids are non-uniform and randomly distributed inside a porous medium, the total pore area in any cross-section is equal to *P*π*r*_1_^2^. Then the average amplitude of air oscillations in pores can be calculated by the formula

(3) $$b_{air\_p}=b_{f}/P.$$

The protocol for each experiment is the following. The temperature of water in the thermostat is set to the temperature, which is close to the ambient temperature. The free surface of 2-propanol in the transparent cell is clearly visible and its distance *h_f_* from the bottom end of the channel/porous medium can be readily obtained by image processing. The camera is controlled by the software Canon EOS Utility which enables to trigger image capture at regular intervals during few hours an experiment lasts, typically each 1 min. The equilibrium regime of the fluid evaporation is typically reached after 1 – 2 hours, and the recording of the free surface begins.

During the experiment, the camera takes a sequence of 300 – 600 photos. We measure the position of the free surface of the fluid relative to the lower boundary of the channel or porous medium on each photo. To do that, we use “Straight” and “Measure” tools of image processing software ImageJ [13].

#### Validation of the method for the determination of molecular diffusion coefficient

As the fluid evaporates through the channel of variable radius or porous medium, its free surface descends at a constant velocity *dh_f_* /*dt*. Recently, Kozlov and Polezhaev [10] stated that knowledge of this velocity is essential to calculate the molecular diffusion coefficient in a channel of variable radius:

(4) $$D\;=\;\rho_{f}\frac{r_{1}^{2}}{r^{2}}\frac{dh_{f}}{dt}\frac{L}{\rho_{0}}.$$

Now, we consider the method of calculating the molecular diffusion coefficient of vapor in air in a porous medium. First, we take into account that vapor diffuses through pores, which total area is equal to *P*π*r*_1_^2^ in each section of a porous medium. Second, we pay attention to the fact that the upper and lower boundaries of a porous medium are connected by tortuous pore paths. Consequently, the diffusion coefficient of 2-propanol vapor in air must be corrected for the tortuosity. The diffusive tortuosity τ is defined as the diffusion coefficient of diffusing species in free fluid or gas, *D*, relative to its value in a porous medium, *D_p_* [14]:

(5) $$\tau \equiv D/D_{p}.$$

Determination of τ is an important practical problem, and there is a large number of studies devoted to the estimation of τ for various porous materials (for example, [15], [16], [17]). The proposed experimental setup can be used to estimate the diffusive tortuosity of porous media. The main idea of our approach is to compare the coefficient of vapor diffusion in air in a porous medium *D_p_* and the coefficient of vapor diffusion obtained from Eq. (4). Taking into consideration comments made above, Fick's law for vapor diffusion in a porous medium can be written in the following form

(6) $$\frac{dm}{dt}=D_{p}P\pi r_{1}^{2}\frac{\rho_{0}}{L}.$$

The mass of evaporated fluid in the glass cell can be evaluated from the equation

(7) $$\frac{dm}{dt}=\rho_{f}\pi r_{1}^{2}\frac{dh_{f}}{dt}.$$

Combining Eqs. (6) and (7), one can obtain the equation for the diffusion coefficient:

(8) $$D_{p}\;=\;\frac{dh_{f}}{dt}\frac{\rho_{f}}{\rho_{0}}\frac{L}{P}.$$

**Fig. 2:**
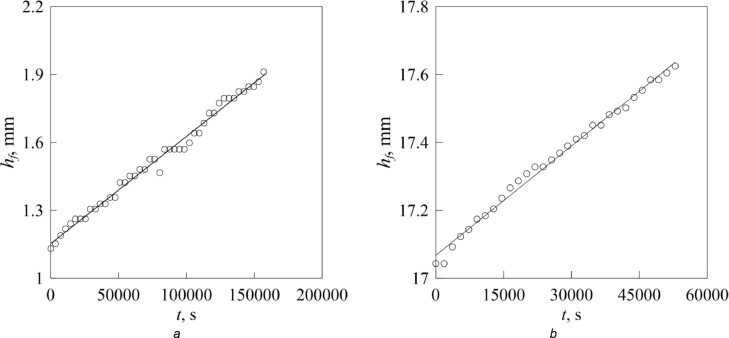
Evolution of the free surface position due to evaporation (without oscillation) in the experiments with the channel of variable radius (*a*) and porous medium consisting of randomly packed hard spheres (*b*). Data in the left graph were first published in [10]; Data in the right graph were first published in [11].

Fig. 2 demonstrates the time evolution of distance *h_f_* in the experiments with the channel of variable radius (*a*) and porous medium consisting of randomly packed hard spheres (*b*). The analysis of experimental data in Fig. 2*a* makes it possible to determine the free surface velocity *dh_f_* /*dt* and then the coefficient of molecular diffusion *D*. The experimental value of the molecular diffusion coefficient obtained from formula (4) *D_ch_* = 13 mm^2^/s is somewhat larger than the reference value *D* = 9.8 mm^2^/s [18]. Substitution of the free surface velocity *dh_f_* /*dt* into Eq. (8) makes it possible to evaluate the coefficient of vapor diffusion in air in a porous medium *D_p_* and then the diffusive tortuosity τ. For the experimental data in Fig. 2*b*, the diffusion coefficient *D_p_* = 7.0 mm^2^/s and the diffusive tortuosity τ*_exp_* = 1.43. Now, we can compare the experimental value τ*_exp_* with the theoretically predicted one. For random packing of monosized particles, the following equation is valid [19]

(9) τ=1−λlnP,

here dimensionless parameter λ depends on the shape of particles and equals 0.49 for beds of spheres [19]. Then, τ = 1.45 favorably agrees with the experimental value τ*_exp_* = 1.43.

#### Validation of the method for the determination of effective diffusion coefficient

The experimental setup also makes it possible to measure mass the transfer of vapor in the presence of air longitudinal oscillations in a channel or a porous medium. As before, we can use Eqs. (4) and (8) to obtain the coefficient of effective diffusion.

**Fig. 3:**
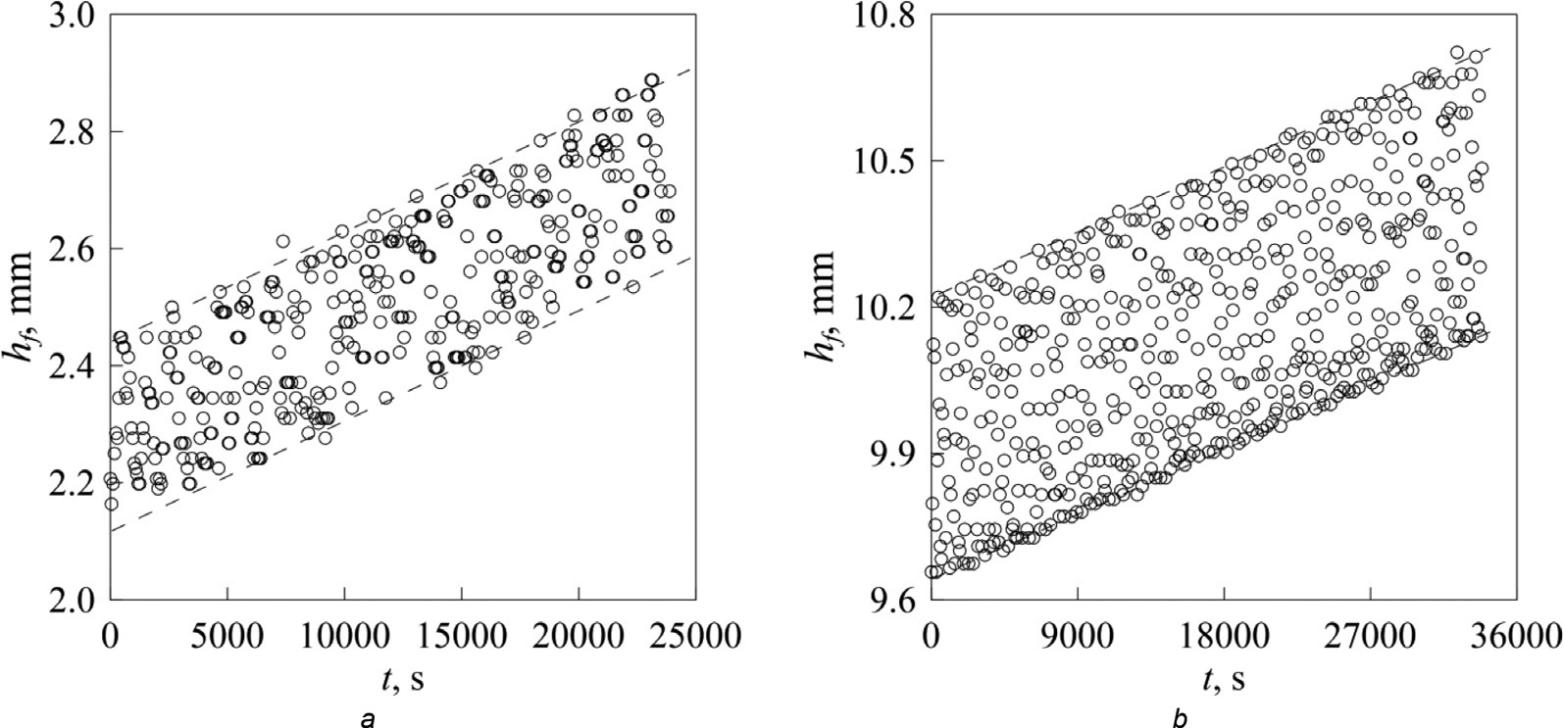
Evolution of the free surface position due to evaporation in the experiments with the channel of variable radius (*a*) and porous medium consisting of randomly packed hard spheres (*b*): *f* = 150 Hz and *b_f_* = 0.14 mm (*a*), *f* = 40 Hz and *b_f_* = 0.29 mm (*b*).

The only change occurs in the measurement of the velocity of the free surface of evaporating fluid *dh_f_* /*dt*. During an experiment with oscillating fluid, the DSLR camera and the digital generator of the harmonic signal are not synchronized and therefore images of the free surface are captured at various phases of the fluid oscillations. So, plotting the free surface positions as a function of time results in a scatter of experimental data points with edges corresponding to the lowermost and uppermost positions of the free surface (Fig. 3). Here, the velocity is determined by the slope of the line drawn along the lowermost or uppermost symbols.

Comparison of experimental data in Fig. 2 and Fig. 3 shows that the diffusion rate in the presence of air oscillations is larger than the coefficient of molecular diffusion.

**Fig. 4:**
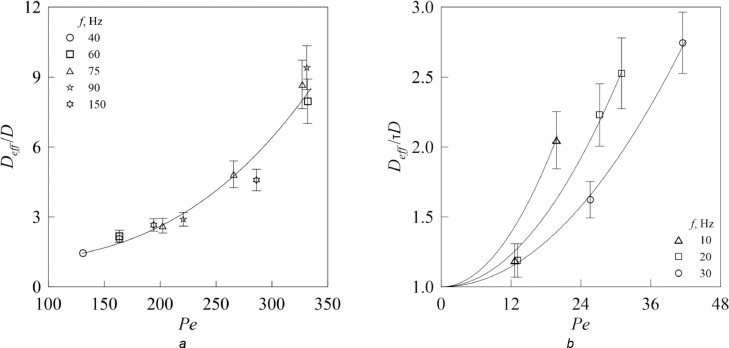
Dimensionless effective diffusion coefficient versus Peclet number for the channel with periodically varying radius (*a*) and porous medium (*b*).

The efficiency of air oscillations can be estimated by calculating the ratio *D_eff_* /*D* in a channel of variable radius (Fig. 4*a*) or *D_eff_* /τ*D* in a porous medium (Fig. 4*b*). It can be seen that the coefficient of effective diffusion *D_eff_* increases with an increase in the Peclet number *Pe* = 2π*fb_air_d*/*D*, here amplitude of air oscillations is determined by the formula (2) or (3), *d* is the diameter of the narrow section of the channel or the diameter of spherical particles composing the porous medium.

### Conclusion

A new technique for measuring the diffusion coefficient of vapor of a volatile liquid in atmospheric air is discussed. We study the evaporation of a volatile liquid in a channel of constant (or variable) radius or a thick layer of a porous medium. The diffusion coefficient is calculated from Fick's law considering that mass of evaporated liquid equals to mass of air diffused through the channel or porous medium.

In the absence of air oscillations, the technique allows measuring the coefficient of molecular diffusion of vapor in air. Perhaps an even more significant result is that a new approach can be used to measure the coefficient of enhanced vapor diffusion in oscillating air which was first discovered in very recent research [10]. Another useful side effect of the new technique is that it makes it possible to calculate the diffusive tortuosity of porous media. For this, it is necessary to measure both the molecular diffusion coefficient of vapor in air in a straight tube and the coefficient of vapor diffusion through the pores and then to calculate τ given by Eq. (5).

The experimental results obtained using the proposed technique are useful for understanding the processes taking place in various technical operations including wood and food drying, drug delivery, etc. Future work will focus on studies of enhanced diffusion in real porous media under ultrasound irradiation.

## Declaration of Competing Interest

The authors declare that they have no known competing financial interests or personal relationships that could have appeared to influence the work reported in this paper.
